# Chemical property based sequence characterization of PpcA and its homolog proteins PpcB-E: A mathematical approach

**DOI:** 10.1371/journal.pone.0175031

**Published:** 2017-03-31

**Authors:** Jayanta Kumar Das, Pabitra Pal Choudhury

**Affiliations:** Applied Statistics Unit, Indian Statistical Institute, 203 B.T Road, Kolkata-700108, West Bengal, India; Tianjin University, CHINA

## Abstract

Periplasmic c7 type cytochrome A (PpcA) protein is determined in Geobacter sulfurreducens along with its other four homologs (PpcB-E). From the crystal structure viewpoint the observation emerges that PpcA protein can bind with Deoxycholate (DXCA), while its other homologs do not. But it is yet to be established with certainty the reason behind this from primary protein sequence information. This study is primarily based on primary protein sequence analysis through the chemical basis of embedded amino acids. Firstly, we look for the chemical group specific score of amino acids. Along with this, we have developed a new methodology for the phylogenetic analysis based on chemical group dissimilarities of amino acids. This new methodology is applied to the cytochrome c7 family members and pinpoint how a particular sequence is differing with others. Secondly, we build a graph theoretic model on using amino acid sequences which is also applied to the cytochrome c7 family members and some unique characteristics and their domains are highlighted. Thirdly, we search for unique patterns as subsequences which are common among the group or specific individual member. In all the cases, we are able to show some distinct features of PpcA that emerges PpcA as an outstanding protein compared to its other homologs, resulting towards its binding with deoxycholate. Similarly, some notable features for the structurally dissimilar protein PpcD compared to the other homologs are also brought out. Further, the five members of cytochrome family being homolog proteins, they must have some common significant features which are also enumerated in this study.

## Introduction

Amino acids play the vital role for determining the protein structure and functions. But it is informative to know how the functionality of the group of proteins is changed while amino acid patterns are getting changed from one protein to another. It becomes quite harder and mostly time consuming to identify the uniqueness of proteins and their functionality from the wet lab experiments while working with complete sequence. In this regard, several techniques have been developed for the analysis of primary protein sequence that is helping the biochemist to work with only specific domain instead of the whole sequence which reduces the experiment time.

Geobacter sulfurreducens is one of the predominant metal and sulphur reducing bacteria [1]. The organism Geobacter sulfurreducens is known to act as an electron donar and participate in redox reaction [2]. Periplasmic c7 type cytochrome A (PpcA) protein along with its four additional homologs (PpcB-E: PpcB, PpcC, PpcD, PpcE) are identified in Geobacter sulfurreducens genome [3–6]. Altogether, five proteins are highly conserved around “heme IV” but are not identical, and mostely differ in two hemes, “heme I” and “heme III” [4]. These two regions are known to interact with its own redox partner. Deoxycholic acid (conjugate base deoxycholate), also known as cholanoic acid, is one of the secondary bile acids, which are metabolic byproducts of intestinal bacteria used in medicinal field and for the isolation of membrane associated proteins [7, 8]. Among the five members of cytochromes c7 family, only PpcA can interact with deoxycholate (DXCA) while its other homologs cannot. While interacting with DXCA, it is observed that few residues are utilized [4, 6, 9]. It would be worthy if the reason of such an amazing difference towards recognizing a single compound can be found through the amino acids sequence viewpoints. Further, one can also see the reason of the structural dissimilarity of PpcD compared to the other homologs [5].

In literature, in-silico techniques have been used to tackle the various problems through the analysis of DNA, RNA and protein sequences in bioinformatics field. Specially, the authors are searching the protein blocks which are highly similar and conserved among the sub-group or entire family members [10–13]. There are twenty standard naturally occurring amino acids which are diverse, arises complexity in the sequences, and have some group specific susceptibility. Various reduced alphabet methods are established which can perform much better in certain conditions [14–17]. Sequence similarity is the most widely reliable strategy that has been used for characterizing the newly determined sequences [18–21]. Finding the functional/structural similarity from homolog sequences with low sequence similarity is a big challenging task in bioinformatics. To tackle this problem, several methods have been introduced that can identify homolog proteins which are distantly distributed in their evolutionary relationships [22–25]. Again, in microRNA field the authors have developed a new identification technique of MicroRNA precursors emphasizing on different data distributions of negative samples [26]. Further, phylogenetic analysis are also studied from different viewpoints to find the evolutionary relationship among various species [27–29]. Some authors have used the statistical tools for sequence alignment, alignment-free sequence comparison and phylogenetic tree [30–33]. Although every amino acid has individual activity, group specific function of amino acid is also obvious. Methods have been introduced for the 2D graphical representation of DNA/RNA or protein sequences [34–40] where methods are based on individual score and position wise graphical representation. So, in this field establishment of a new methodology is always welcome with distinct findings. Combining with various features for DNA, RNA and protein sequence a web server called Pse-in-One (http://bioinformatics.hitsz.edu.cn/Pse-in-One/home/) is developed [41] which is user friendly and can be modified by users themselves. Recently, the authors have classified the twenty standard amino acids into the eight chemical groups and have found some group and/or family specific conserved patterns which are involved in some functional role specially in motor protein family members [17].

In this study, the previously defined method [17] of reduced alphabets are used as an application into the cytochrome c7 family protein members. We introduced a new method of phylogenetic analysis based on chemical group dissimilarity of amino acids. In addition, we build the graph from primary protein sequence. In the designing of graph, we have designated the various chemical groups of amino acids as thevertices in the graph. The primary protein sequence is read as consecutive order pairs serially from first amino acid to the end of sequence, and each order pair is nothing but a connected edge between the two nodes where nodes in the graph are involved with different chemical groups of amino acids. The graph is drawn for every individual protein sequence and we look for various unique edges/cycles among the entire family members. So any unique findings from the graph may be hypothesized as having a significant functional role in the primary protein sequence. Because the variation in the graph is directly affected by the amino acid residues in some specific domain where a change of chemical group has taken place. We highlight all the significant points which are differing from one sequence to other. Further, working with reduced alphabets and designing the graph require less complexity and easy visualization even if working with the larger sequences.

## Methods and materials

### Order pair directed graph

A directed graph *G* = (*V*, *E*) is a graph which consists of a set of vertices denoted by *V* = {*V*_1_, *V*_2_, …, *V*_*i*_}, and a set of connected edges denoted by *E* = {*E*_1,1_, *E*_1,2_, …, *E*_*i*, *j*_} where an edge *E*_*i*, *j*_ exists if the corresponding two vertices *V*_*i*_ and *V*_*j*_ are connected and the direction of edge is from the vertex *V*_*i*_ to the vertex *V*_*j*_. From the graph, various graph theoretic properties like edge connectivity, cycles, graph isomorphism etc. can be investigated to differentiate the graphs.

Given an arbitrary amino acids sequence, it is first transformed into the numerical sequence as described previously where amino acids are categorized into eight chemical groups according to the side chain/chemical nature of the amino acids [17]. The transformation is done using the following rules (Eq 1) as per the classification. If a particular amino acid is read as *A*_*i*_, then the corresponding transformed group is *G*_*k*_ and the numerical value *k* is defined by the following Eq (1).

$$\begin{array}{c} G_{k}=\left\{\begin{array}{cc} G_{1}/1 & :ifA_{i}\in \left\{D,E\right\}\\ G_{2}/2 & :ifA_{i}\in \left\{R,H,K\right\}\\ G_{3}/3 & :ifA_{i}\in \left\{Y,F,W\right\}\\ G_{4}/4 & :ifA_{i}\in \left\{I,L,V,A,G\right\}\\ G_{5}/5 & :ifA_{i}\in \left\{P\right\}\\ G_{6}/6 & :ifA_{i}\in \left\{M,C\right\}\\ G_{7}/7 & :ifA_{i}\in \left\{S,T\right\}\\ G_{8}/8 & :ifA_{i}\in \left\{Q,N\right\} \end{array}\right. \end{array}$$ (1)

Here, *G*_1_, *G*_2_, …, *G*_8_ are the Acidic, Basic, Aliphatic, Aromatic, Cyclic, Sulfur Containing, Hydroxyl Containing and Acidic Amide groups respectively [17]. The eight numerical values are considered as the vertices of the graph *G* i.e. *V*_*i*_ ∈ {1, 2, …8}. Algorithm 1 is used to generate the directed graph from the primary protein sequence using MATLAB16b software. Here, we obtain the graph which is the order pair digraph because an edge is constructed through the pair (source node, target node) which is obtained from the consecutive order pair list of amino acids in the primary protein sequence. So given an arbitrary amino acid sequence, we can find an order pair directed graph having at most eight vertices/nodes.

**Algorithm 1:**

**Input:** Primary protein sequence (A) of length *L* (# Amino Acids) where *A* = *A*_1_, *A*_2_, …, *A*_*L*_.

**Output:** An adjacency matrix and the corresponding order pair directed graph.

Define a null matrix (M) of size 8 by 8;

Define a 1-D array (T) of size L,;

**for** *i* = *1:length(A)* **do**

 Read *A*_*i*_;

 Find *X* as the chamical group number of *A*_*i*_ uisng Eq (1);

 *T*(*i*) = *X*;

**end**

**for** *i* = *1:length(T)-1* **do**

 Read: (*T*(*i*), *T*(*i* + 1));

 **if** *M*(*T*(*i*), *T*(*i* + 1)) == *0* **then**

  *M*(*T*(*i*), *T*(*i* + 1)) = *M*(*T*(*i*), *T*(*i* + 1)) + 1;

 **end**

**end**

k = 1;

**for** *i* = *1:length(T)* **do**

 **for** *j* = *1:length(T)* **do**

  **if** *M(i, j)≠ 0* **then**

   s(k) = i;

   t(k) = j;

   k = k + 1;

  **end**

 **end**

**end**

G = digraph(s, t);

plot(G);

### Phylogenetic tree formation

The phylogenetic tree is an acyclic graph showing the evolutionary relationship among the various biological species based on their genetic closeness. Although various phylogenetic tree methods have already been studied, based on chemical nature of amino acids are not yet explored in the literature as per our knowledge. Our method of phylogenetic tree formation used the dissimilarity matrix which is obtained for every pair of sequence on the basis of chemical group specific score of amino acids. So this method is completely alignment free and requires less computational complexity.

Firstly, we calculate the percentage of occurrence of amino acids from each chemical group using the following equation Eq (2). If there are *n* number of sequences which are denoted as *S*_1_, *S*_2_, …*S*_*n*_, then the corresponding length of the sequences are denoted as *L*_1_, *L*_2_, …*L*_*n*_. And a particular sequence *S*_*i*_ is read as $S_{i}=S_{i}^{1},S_{i}^{2},...S_{i}^{L_{i}}$. For the sequence *S*_1_, the first amino acid is read as $S_{1}^{1}$, the second amino acid is read as $S_{1}^{2}$ and so on. For each *G*_*k*_ group and a particular sequence *S*_*i*_, we count the total number of amino acids *S*_*i*_(*T*_*k*_) and score per hundred *S*_*i*_(*G*_*k*_) on using the following Eqs (2) and (3) respectively.

$$\begin{array}{c} S_{i}\left(T_{k}\right)=\sum_{l=1}^{L_{i}}S_{i}^{l} \end{array}$$ (2)

where

$$\begin{array}{c} S_{i}^{l}=\left\{\begin{array}{cc} 1 & \;\text{if}\;S_{i}^{l}\in G_{k}\\ 0 & \;\text{otherwise} \end{array}\right. \end{array}$$

$$\begin{array}{c} S_{i}\left(G_{k}\right)\left(\%\right)=\frac{S_{i}\left(T_{k}\right)}{L_{i}}\times 100 \end{array}$$ (3)

For example, if the primary protein sequence length is 80 aa, out of which 20 aa are from acidic group i.e. *G*_1_, then the score per hundred of the acidic group is $(\frac{20}{80}\times 100)=25\%$.

Secondly, we measure the dissimilarity measure for every possible pair of sequence. The dissimilarity of two sequences *S*_*i*_ and *S*_*j*_ is denoted as $D_{S_{i}}{{}_{,}}_{S_{j}}$. For each group *G*_*k*_, we count the percentage of amino acid differences of the two sequences taking the mod value of the score obtained on using Eq (4). This is done for all the respective eight chemical groups and all the values are added. Finally, we get the dissimilarity matrix D of size *n* by *n* as shown below.

$$\begin{array}{c} D_{ij}=D_{S_{i},S_{j}}=\sum_{k=1}^{8}∥S_{i}\left(G_{k}\right)-S_{j}\left(G_{k}\right)∥ \end{array}$$ (4)

$$\begin{array}{c} D\left(n,n\right)=\left(\begin{array}{cccc} D_{11} & D_{12} & \cdots & D_{1n}\\ D_{21} & D_{22} & \cdots & D_{2n}\\ \vdots & \vdots & \ddots & \vdots \\ D_{n1} & D_{n2} & \cdots & D_{nn} \end{array}\right) \end{array}$$

To draw the phylogenetic tree, we use the nearest distance (single linkage) method. The pair wise distances are the entities of the obtained dissimilarity matrix and the whole procedure is written in MATLAB 2016b software.

### Data set specification

Five homologous triheme cytochromes (PpcA-E) are identified in G. sulfurreducens periplasm and gene knockout studies revealed their involvement in Fe(III) and U(VI) extracellular reduction [1, 2]. Cytochromes have been thoroughly studied for laboratory experiments because of their small size (about 90 amino acids). Table 1 shows the gene name, accession number, protein name, length (#amino acids). The primary protein sequences are collected from http://www.uniprot.org/.

**Table 1:** Details of five members of cychrome c7 family in Geobacter sulfurreducens.

Seq. Nos.	Gene Name	Length (aa)	Chain domain	Accession Number	Protein Names
1	PpcA	91	21-91	Q8GGK7	Cytochrome c
2	PpcB	91	21-91	Q74G83	Cytochrome c
3	PpcC	95	21-95	Q74G82	Cytochrome c
4	PpcD	92	21-92	Q74ED8	Cytochrome c
5	PpcE	90	21-90	Q74CB4	Cytochrome c

## Results and discussion

### Sequence identity and the phylogenetic tree

Firstly, our analysis is directed to measure the primary protein sequence for every member. We obtain the percentage identity matrix of every pair of sequences (Table 2) which is exported from ClustalW. It is observed that sequences are at least 47% similar. The maximum similarity is 76% which is found between PpcA and PpcB. If we consider the PpcA sequence which shows the minimum of 50% similarity with PpcE and the maximum of 76% similarity with PpcB, we are not able to differentiate the PpcA from other homologs on using the similarity percentage.

**Table 2:** Percentage identity matrix of every pair sequence of cytochrome c7 family members.

**Seq. Nos.**	**1**	**2**	**3**	**4**	**5**
1	100.00	75.82	58.24	63.33	50.00
2		100.00	53.85	64.44	52.22
3			100.00	48.89	46.67
4				100.00	47.78
5					100.00

Secondly, we count rate of occurrence (frequancy of amino acids) of every individual amino acid of the respective five sequences which are shown in Table 3. Then, we look for chemical group specific frequency for every sequence shown in Table 4 using Eq (3).

**Table 3:** Lists the total number of occurance of each respsctive amino acids of five sequences.

Seq. Nos.	D	E	R	H	K	Y	F	W	I	L	V	A	G	P	M	C	S	T	Q	N
1	4	6	0	6	19	0	4	0	3	4	5	10	11	4	3	7	2	1	1	1
2	3	5	0	6	15	0	4	0	3	5	3	11	10	3	4	6	2	8	1	2
3	5	4	5	7	9	1	3	0	8	4	4	9	15	5	3	6	1	5	1	0
4	3	6	1	7	15	0	2	1	2	5	6	13	12	1	3	7	0	6	0	2
5	2	4	6	6	10	1	5	0	4	5	6	8	8	3	2	6	4	8	0	2

**Table 4:** Number count of every chemical group in percentage wise of cytochrome family members.

Seq. Nos.	G1(%)	G2(%)	G3(%)	G4(%)	G5(%)	G6(%)	G7(%)	G8(%)
1	10.9890	27.4725	4.3956	36.2637	4.3956	10.9890	3.2967	2.1978
2	8.7912	23.0769	4.3956	35.1648	3.2967	10.9890	10.9890	3.2967
3	9.4737	22.1053	4.2105	42.1053	5.2632	9.4737	6.3158	1.0526
4	9.7826	25.0000	3.2609	41.3043	1.0870	10.8696	6.5217	2.1739
5	6.6667	24.4444	6.6667	34.4444	3.3333	8.8889	13.3333	2.2222

**Fig 1:**
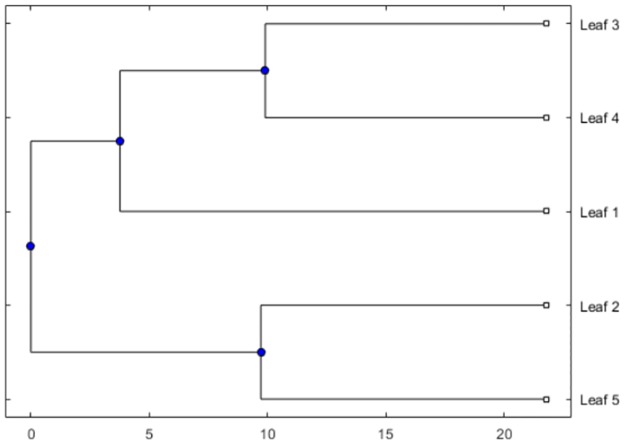
The phylogenetic tree of the PpcA-E five members. The phylogenetic tree is obtained based on the pair wise dissimilarity matrix (Table 5), and the method is used nearest distance (single linkage method) in MATLAB 2016b. The leaves 1-5 are the corresponding PpcA-E five members respectively.

Now, we obtain the dissimilarity score of all possible two sequences (using Eq (4)). Say for an example, we compare the Seq. no. 1 and Seq. no. 2, we get the difference for Acidic group is 2.1978 (10.9890-8.7912), Basic group is 4.3956 (27.4725-23.0769) and so on (from Table 4). Total score after summing the eight groups is 17.5824 which measures the dissimilarity percentage of the said two sequences. Similar results we get for all other pairs which are shown in Table 5. This table shows the biological distances between each pair of sequences. From this pair wise distance matrix, the phylogenetic tree is constructed as shown in Fig 1, also discussed in method Section. Based on the phylogenetic tree of five members, we find that the PpcA and PpcD, PpcB and PpcE are mostly closed with regards to the frequency of amino acids of respective eight chemical groups.

**Table 5:** Pair wise dissimilarity matrix for every pair of sequence of cytochrome c7 family members.

**Seq. Vs. Seq.**	**1**	**2**	**3**	**4**	**5**
1	0	17.5824	19.4563	16.5313	24.6642
2		0	19.1787	18.1080	12.0391
3			0	11.8535	25.9649
4				0	25.0242
5					0

From Fig 1 it is not obvious that PpcA differs from other homologs, but if we go through the dissimilarity matrix (Table 5), we find some variations. Here, it is observed that PpcA differs by minimum of 16.5313% with PpcD, whereas for other homologs minimum dissimilarity is found for PpcD with PpcC which is 11.8535%. Therefore among all the pairs, the high dissimilarity of PpcA shows its uniqueness compared to its homologs. If we have a closer look into the list of amino acids, it is observed that the amino acids D, E, H, K, F, I, L, V, A, G, P, M, C, T are present among all the sequences. Other amino acids are not common to all the member sequences. Therefore, on the basis of chemical groups, all the amino acids from Acidic, Aliphatic, Cyclic and Hydroxyl containing groups are present. It is observed that the Acidic, Basic and Hydroxyl containing groups percentage distinctly differ while compared PpcA with other homologs. Further, it is observed that only one Proline(P) from Cyclic group is present in PpcD while in other homologs, Proline (P) is present at least 3 times. And another important observation is that the amino acid Tryptophan (W) from Aromatic group is present only in PpcD sequence.

### Graph based analysis

**Fig 2:**
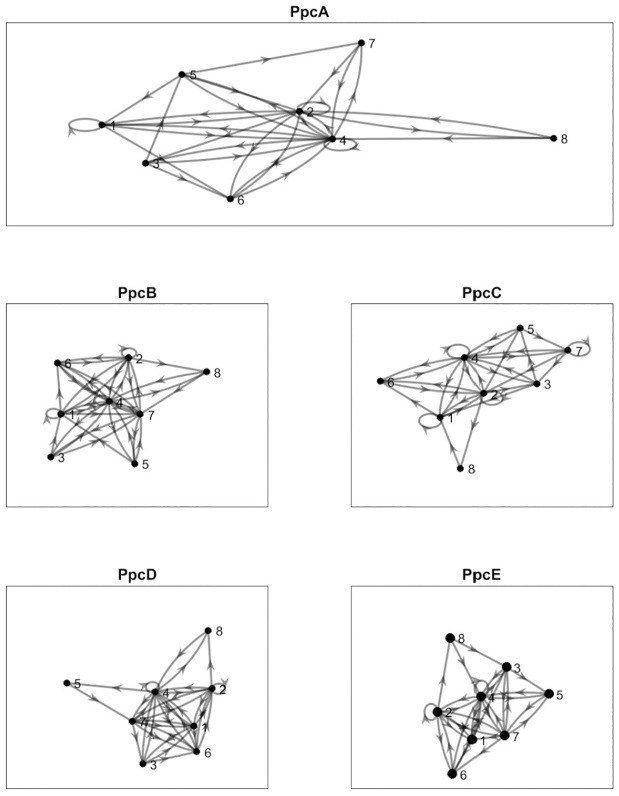
Order pair directed graph (Digraph) of PpcA-E five members. There are eight nodes (1, 2 … 8) for each graph which are the representative of corresponding eight chemical groups (Eq 1).

For every member of cytochrome c7 family, we draw a order pair directed graph using Algorithm 1 which are shown in Fig 2.

There are maximum of eight possible nodes and the various directed edges among the nodes. We try to highlight the connected edges that show the uniqueness, specially in between the PpcA and its homolog members and PpcD with other members separately as well as commonality to all members. Details of the edge connectivity information for PpcA and its homologs are shown in Table 6. We say two nodes (direction is from row to column) are connected or present if the cell symbol is 1, not present if the cell symbol is 0, and common to all the members if the cell symbol is *. An edge between two nodes (in order) is basically a pattern (two distinct nodes or two distinct amino acids from two different chemical groups) of length 2. We find two particular edges, one edge (82) is present only in PpcA sequence (approx. residues 41-42, S1 Table) that is not found in other member sequences, and one edge (73) which is present in PpcB-E sequences (approx. residues 25-36, S1 Table), but this edge is not present in PpcA sequence. While considering all the members, we find many edges which are common to all. Further, PpcD is structurally dissimilar among the homologs [4]. While looking into the order pair directed graph, we find only one variation i.e. there is an edge (54) node 5 to node 4 among the PpcA-C and PpcE sequences which is not observed in PpcD (Table 7). This node transition where amino acid changes Proline(P) to Glycine (G) for PpcA-C and PpcE and for PpcD this transition is from Glycine (G) to Glycine (G), located in approximately residues 54-55 (S1 Table). Again existence of edges between any two nodes either common to all or individual member specific have some significant role in the primary protein sequences. Because node to node connectivity is the point of changes from one chemical group to the other in the primary protein sequence positions and this could be the effective characteristic for the structural or functional variation of proteins.

**Table 6:** Existance of unique edges comparison between PpcA and PpcB-E groups obtained from directed graph (Fig 2).

	PpcA								PpcB-E							
Node Vs. Node	1	2	3	4	5	6	7	8	1	2	3	4	5	6	7	8
**1**		*		*		*				*		*		*		
**2**	*	*		*		*		*	*	*		*		*		*
**3**				*								*				
**4**	*	*	*	*	*	*	*		*	*	*	*	*	*	*	
**5**							*								*	
**6**		*		*						*		*				
**7**		*	0	*			*			*	1	*			*	
**8**		1								0						

**Table 7:** Existance of unique edges comparison between PpcD and PpcA-C, PpcE groups obtained from directed graph (Fig 2).

	PpcD								PpcA-C, PpcE							
Node Vs. Node	1	2	3	4	5	6	7	8	1	2	3	4	5	6	7	8
**1**		*		*		*				*		*		*		
**2**	*	*		*		*		*	*	*		*		*		*
**3**				*								*				
**4**	*	*	*	*	*	*	*		*	*	*	*	*	*	*	
**5**				0			*					1			*	
**6**		*		*						*		*				
**7**		*		*			*			*		*			*	
**8**																

**Fig 3:**
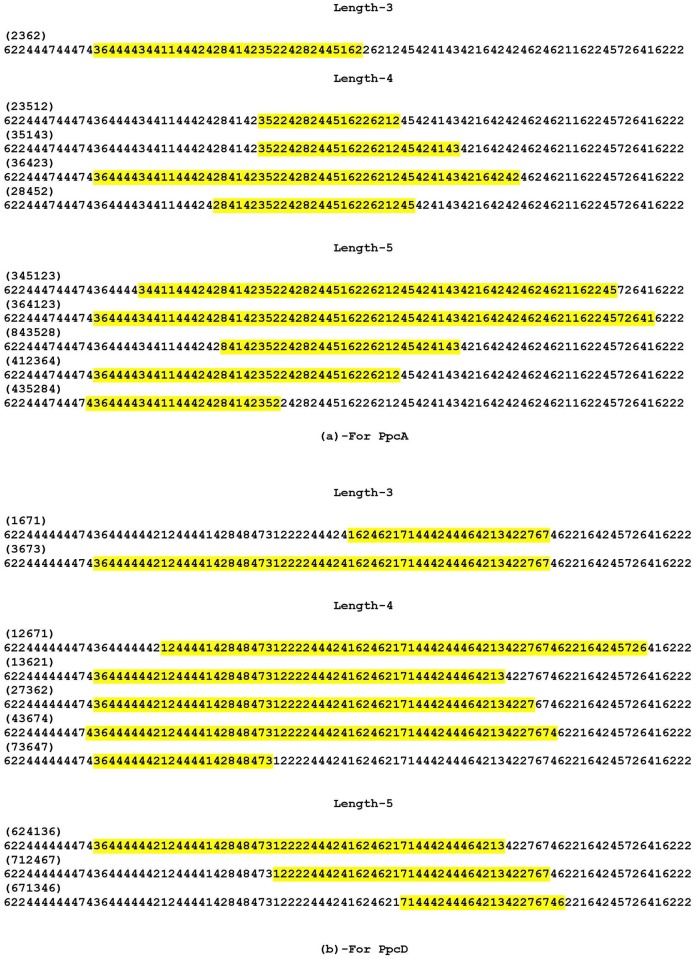
Unique cycles involving various sub-domains for PpcA and PpcD.

Although few residues are being responsible while interacting with DXCA, the neighbouring residues of amino acids must be having a role for their unique characteristics. So the subdomain identification involving with different unique cycles would be worth mentioning in this regard. Here, we have calculated the various cycles of length *C*_*L*_ (3 ≤ *C*_*L*_ ≤ 6) for group specific and individual member specific which are shown in in S2 Table. Say for an example, the cycle 7216457 of length 6 i.e. the directed edges are 7 → 2 → 1 → 6 → 4 → 5 → 7. For completing this cycle a particular subdomain is responsible. Interestingly, we find various unique cycles for PpcA, PpcD and PpcB-E. So there are some unique cycles which are distinctly present for PpcA and its homolog proteins and vice versa. There are some unique cycles which are present in PpcD, but no unique cycle is present for PpcA-C and PpcE. Highlighiting the sub-domain for some of the unique cycles of length 3, 4 and 5 are shown in Fig 3(a) for PpcA and Fig 3(b) for PpcD. From Fig 3, the cycle (2362) of length 3 whose sub-domain residues are within 13 to 48, that is the numerical sequence is 36…62 from Fig 3(a). One can see the corresponding amino acids residues from S1 Table. For some cycles, there is a possibility of different sub domains because some edges are repeating more than once in the different positions of the sequence that can be counted for the same cycle. Similarly, on varying the cycle length, we get different sub-domains or amino acid residues. These sub-domain findings might be of immense help to the Bio-chemists for the understanding of physicochemical nature and the unique activity of various proteins.

### Comparison based on conserved patterns

**Fig 4:**
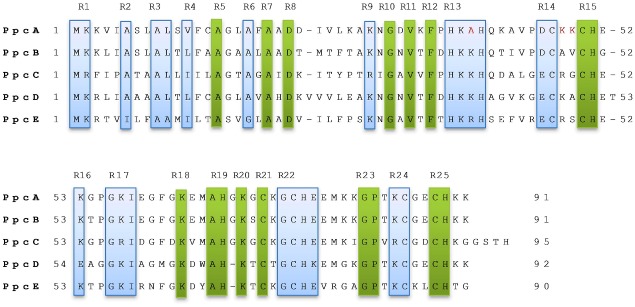
Various conserved regions for the five homolog members of PpcA family. The boxes with highlighted regions are having the chemical group specific conserved and other highlighted regions are conserved based on individual amino acid.

We take all the five sequences of PpcA-E members, obtain the alignment sequence from ClustalW2. The alignment figure is shown Fig 4. We mark the various blocks as R1, R2…R16 which are conserved. Rectangular with highlighted regions are chemically conserved, and only highlighted regions are conserved based on individual amino acid. We find two highly conserved regions R13 and R22 which are having some variations. The first region (R13) is with 4 residues block (HKK/RH or 2222) among the members PpcB-E where all the amino acids from Basic group, but in PpcA this block is HKAH or 2242 i.e. the 3rd position K/R is replaced by Aliphatic amino acid Alanine (A). The second region (R22) is GCHE/K or 4622/1 where 4th position amino acid is either from Acidic or Basic group i.e. both fall under Charge group. If we look into the PpcA sequence some dissimilarities are found in “Heme I” region [3–5]. The two consecutive amino acids between regions R14 and R15 in PpcA is KK (from Basic group), but for PpcB-E only one amino acid is from Basic group. Previously it is observed that PpcD is structurally dissimilar [5] and the authors have shown that there is an addition of amino acid Threonine (T) for PpcD sequence after the R15 region in Fig 4. But, from figure we can see that another one amino acid Valnine (V) insertion is viewed in region of R8 and R9. Besides, various patterns which are common to PpcA, but not in PpcB-E and vice versa shown in Table 8 with bold color. For the pattern “624621” which is located with the combined regions of R21 and R22 (“heme III” region), there is a change of amino acid Threonine (T) for PpcD and Lysine (K) for others. Apart from these, we find an amino acid deletion both for the PpcD and PpcE before the “Heme III” region. Further, on combining the regions R6, R7 and R8 (pattern “44441”), the change for PpcA sequence is Phenylalanine (F) which is from Arometic group whereas other sequences are from Aliphatic group, and the change for PpcD sequence is Histidine (H) which is from Basic group whereas other sequences are also from Aliphatic group. Again the region between R17 and R18 PpcD contains the amino acid Methionine (M) from the Sulfur containing group while the other homologs contain Phenylalanine (F) from Aromatic group. Altogether, group specific changes have significant role towards the binding with the DXCA for PpcA and the structural dissimilarity of PpcD.

**Table 8:** Conserved chemical patterns among PpcA-E members.

Seq. Nos.	Patterns		
	Pos.-Seq.	Pos.-Seq.	Pos.-Seq.
	624621	44441	2222
1	71-CKGCHE	18-A**F**AAD	37-HK**A**H
2	71-CKGCHE	18-ALAAD	37-HKKH
3	71-CKGCHE	18-AGAID	37-HKKH
4	48-C**T**GCHK	24-AVA**H**D	38-HKKH
5	70-CKGCHE	18-GLAAD	37-HKRH

## Conclusion

In this work, we have presented the sequence based characterization of cytochromes c7 family members. We specifically emphasize the distinguished features of PpcA and PpcD compared to the other homologs. Although the study suggests that percent identity among the five members varies between 46% and 75%, on the basis of chemical groups these are shown between 75% and 89%. We highlight some of the chemical groups and their percentage that can distinguish PpcA and PpcD. The dissimilarity features of PpcA may play significant role towards its binding with DXCA. Similar is the case that may happen for PpcD for its structural dissimilarity. Our proposed graph theoretic model can easily show the instant change of amino acids from one group to the other in the sequences. Further, the unique cycles for PpcA and PpcD may expose their outstanding nature. And finally from the alignment graph, chemically conserved regions are highlighted. We observe some special patterns where amino acid(s) from some of the sequences are abruptly changed. All the cases will provide the features for PpcA and PpcD that would explain their unique functionality and/or structural dissimilarity.

It may be noted that there are some existing methodologies [11, 14, 16, 20, 22, 25, 30] which would reflect the sequence pattern information or key features of the observed sequence. Many characteristics of the DNA, RNA and protein sequences can be found out from the web servers and standalone existing tools, one of the important web servers in this regard is defined in [41]. We look at the problem in a different manner, one dealing with embedded chemical properties of amino acids and various mathematical structures. In general, methodology defined in this article is very easy to implement to get the unique features of observed sequences. So, collectively our methodology will add to be combined for the machine learning algorithms to develop refined computational predictors. Hence, the use of reduced alphabets (amino acids) technique involving mathematical basis with the embedded chemical properties of amino acids will be very much useful for the protein homology detection.

## Supporting information

S1 Table Amino acids and transformed numerical sequence based on eight chemical groups for c7 five members. (PDF)

S2 Table Unique cycles for PpcA-E, PpcA, PpcB-E, PpcD. These cycles are involved in various sub-domains, some of which are shown in Fig 3.
(PDF)
