# Supplementary material for: Chemical property based sequence characterization of PpcA and its homolog proteins PpcB-E: A mathematical approach
Source: PLoS One. 2017 Mar 31;12(3):e0175031. doi: 10.1371/journal.pone.0175031 (PMC5376323; doi:10.1371/journal.pone.0175031)
Supplement: S1 Table — (PDF) [file pone.0175031.s001.pdf]

S1 Table. Amino acids and transformed numerical sequence based on eight chemical groups for c7 five members.

>PpcA<br>MKKVIASLALSVFCAGLAFAADDIVLKAKNGDVKFPHKAHQKAVPDCKKCHEKGPGKIEGFGKEMAHGKGCKG<br>CHEEMKKGPTKCGECHKK<br>622444744474364444344114444242841423522428244516222621245424143421642424624<br>621162245726416222
>PpcB<br>MKKLIASLALTLFAAGAALAADTMTFTAKNGNVTFDHKKHQTIVPDCAVCHGKTPGKIEGFGKEMAHGKSCKG<br>CHEEMKKGPTKCGECHKK<br>622444744474344444444176737428484731222287445164462427542414342164242762 4<br>621162245726416222
>PpcC<br>MRFIPATAALLIILAGTAGAIDKITYPTRIGAVVFPHKKHQDALGECRGCHEKGPGRIDGFDKVMAHGKGCKG<br>CHEEMKIGPVRCGDCHKGGSTH<br>623454744444444474444124735724444435222281444162462124542414312464242462 4<br>62116244542641622447 72
>PpcD<br>MKRLIAAAALTLFCAGLAVAHDKVVVLEAKNGNVTFDHKKHAGVKGECKACHETEAGGKIAGMGKDWAHKTCT<br>GCHKEMGKGPTKCGECHKK<br>622444444474364444442124444142848473122224442416246217144424446421342276 7<br>4622164245726416222
>PpcE<br>MKRTVILFAAMILTASVGLAADVILFPSKNGAVTFTHKRHSEFVRECRSCHEKTPGKIRNFGKDYAHKTCKGC<br>HEVRGAGPTKCKLCHTG<br>622744434464474744444144435728444737222271342162762127542428342134227624 6<br>21424445726246274
