# Supplementary material for: Chemical property based sequence characterization of PpcA and its homolog proteins PpcB-E: A mathematical approach
Source: PLoS One. 2017 Mar 31;12(3):e0175031. doi: 10.1371/journal.pone.0175031 (PMC5376323; doi:10.1371/journal.pone.0175031)
Supplement: S2 Table — These cycles are involved in various sub-domains, some of which are shown in Fig 3. (PDF) [file pone.0175031.s002.pdf]

S2 Table. Unique cycles for PpcA-E, PpcA, PpcB-E, PpcD.

Member /Group	Cycles of Length 3
**PpcA-E**	4214 4124 2412 2142 1421 1241 6216 2162 1621 6416 4164 1641 6426<br>6246 4624 4264 2642 2462 7247 4724 2472 7457 5745 4574
**PpcA**	6236 3623 2362
**PpcB-E**	7347 4734 3473
**PpcD**	7167 6716 1671 7367 6736 3673
	**Cycles of Length 4**
**PpcA-E**	64216 64126 62416 62146 46214 42164 41624 41264 26412 24162 21642 21462<br>16421 16241 14621 12641 72147 47214 21472 14721 72457 57245 45724 24572<br>72647 64726 47264 26472
**PpcA**	51235 35123 23512 12351 51435 43514 35143 14351 64236 42364 36423 23642<br>84528 52845 45284 28452
**PpcB-E**	73457 57345 45734 34573
**PpcD**	62136 36213 21362 13621 71267 67126 26712 12671 64136 41364 36413 13641<br>71367 67136 36713 13671 71467 67146 46714 14671 73627 62736 36273 27362<br>74367 73647 67436 64736 47364 43674 36743 36473
	**Cycles of Length 5**
**PpcA-E**	721457 572145 457214 214572 145721 721647 647216 472164 216472 164721 726457 645726<br>572645 457264 264572
**PpcA**	514235 512435 512345 451234 435124 423514 351423 351243 345123 243512 235142 234512<br>142351 124351 123451 641236 412364 364123 236412 123641 623516 516235 351623 235162<br>162351 643516 516435 435164 351643 164351 645236 523645 452364 364523 236452 843528<br>528435 435284 352843 284352 723647 647236 472364 364723 236472
**PpcB-E**	No
**PpcD**	642136 624136 421364 413624 364213 362413 241362 213642 136421 136241 736217 721367<br>713627 672136 627136 621736 367213 362713 362173 271362 217362 213672 173621 136721<br>136271 714267 712467 671426 671246 467124 426714 267142 246712 142671 124671 741367<br>736417 714367 713647 713467 674136 671436 671346 647136 641736 471364 467134 436714<br>417364 413674 367413 367143 364713 364173 346713 173641 143671 136741 136471 134671<br>743627 736427 736247 724367 672436 642736 627436 624736 473624 436724 436274 427364<br>367243 364273 362743 362473 274362 273642 247362 243672 736457 645736 573645 457364<br>364573
	**Cycles of Length 6**
**PpcA-E**	7216457 6457216 5721645 4572164 2164572 1645721
**PpcA**	1236451 1264351 1284351 1462351 1472351 1623451 1624351 1642351 2345162 2351462 2351472 2351642<br>2364512 2364572 2435162 2643512 2843512 3451623 3512643 3512843 3514623 3514723 3516243 3516423<br>3645123 3645723 4235164 4351264 4351284 4351624 4512364 4516234 4572364 4623514 4723514 5123645<br>5126435 5128435 5146235 5147235 5162345 5162435 5164235 5723645 6234516 6235146 6243516 6423516<br>6435126 6451236 6457236 7235147 7236457 8435128
**PpcB-E**	No
**PpcD**	1243671 1273641 1284671 1342671 1346721 1362471 1362741 1362841 1364271 1364571 1364721 1367241 1367421<br>1436271 1436721 1473621 1736241 1736421 1743621 2134672 2136472 2136742 2143672 2147362 2173642 2174362<br>2413672 2417362 2436712 2457362 2471362 2671342 2713642 2714362 2736412 2741362 2841362 2843672 2846712<br>2847362 3426713 3467213 3621473 3621743 3624173 3624573 3624713 3627143 3627413 3628413 3628473 3641273<br>3642173 3642713 3645713 3647213 3671243 3672143 3672413 3672843 3674213 4127364 4136274 4136284 4136724<br>4173624 4213674 4217364 4267134 4271364 4362174 4362714 4367124 4367214 4367284 4571364 4573624 4671284<br>4672134 4713624 4721364 4736214 4736284 5713645 5736245 6214736 6217436 6241736 6245736 6247136 6271436<br>6274136 6284136 6284736 6412736 6421736 6427136 6457136 6472136 6712436 6712846 6713426 6721346 6721436<br>6724136 6728436 6742136 7124367 7128467 7134267 7136247 7136427 7136457 7143627 7213467 7213647 7214367<br>7241367 7284367 7362147 7362417 7362457 7362847 7364127 7364217 7413627 7421367 7436217 8413628 8436728<br>8467128 8473628
